# In vitro and in vivo antimutagenic activity of *Echinops spinosus* crude extract and its aqueous fraction in mouse bone marrow and spleen

**DOI:** 10.1186/s41021-025-00341-z

**Published:** 2025-11-05

**Authors:** Kawthar A. Diab, Ayman A. Farghaly, Entesar E. Hassan, Maha A. Fahmy, Emad M. Hassan, Zeinab M. Hassan

**Affiliations:** 1 Genetics and Cytology Department, National Research Centre (NRC), 33 El-Bohouth st, Dokki, Cairo, 12622 Egypt; 2 Medicinal and Aromatic Plants Research Department, National Research Centre (NRC), 33 El-Bohouth st, Dokki, Cairo, 12622 Egypt; 3 Natural Compounds Department, National Research Centre (NRC), 33 El-Bohouth st, Dokki, Cairo, Egypt

**Keywords:** *Echinops spinosus*, Ethyl methanesulfonate, Genotoxicity; chromosomal aberration; comet assay, Micronucleus, Mice

## Abstract

**Background:**

*Echinops spinosus* (ES), known as spiny globe thistle, has been widely used in traditional medicine to treat various ailments, such as splenic and renal disorders. However, the genoprotective effect of ES has not been examined previously. This report assessed the in vitro and in vivo genoprotective effects of crude extract of *Echinops spinosus* (CEES) and its aqueous fraction (AFES) against ethyl methanesulfonate (EMS) in mice. This study applied a battery of genotoxic endpoints, including chromosomal aberrations (CAs), the comet assay, and the micronucleus (MN) assay. Further, GC-MS and HPLC analyses were employed to identify the primary and secondary metabolites in the plant samples, respectively. Total polyphenol and flavonoid contents (TPC and TFC) were also colorimetrically measured. In vitro experiments were conducted using cultured primary mouse bone marrow and spleen. These cells were treated with two concentrations of CEES or AFES (250 and 500 µg/mL; for 24 h), followed by EMS treatment (300 µg/mL; for two hours) before the harvest. For the in vivo experiments, mice were orally administered CEES and AFES (250, 500 mg/kg; for 7 days), with or without intraperitoneal injection with EMS (300 mg/kg; for 24 h).

**Results:**

GC-MS analysis demonstrated 25 primary metabolites in AFES, and the nitrogenous compound bis(trimethylsilyl) ethylamine was the main constituent. HPLC analysis reported 17 and 14 secondary compounds in CEES and AFES, respectively, in which chlorogenic acid was the main constituent in both samples. Colorimetric analysis showed that CEES exhibited higher TPC and TFC compared to AFES. Genotoxic results showed that EMS increased the levels of CAs and comet tail formation in vitro bone marrow and splenic cultures. Further, EMS caused chromosomal damage, as indicated by a significant increase in the frequency of CAs and MN in vivo mouse bone marrow cells. Supplementation with CEES and AFES alleviated chromosomal and DNA damage induced by EMS, and this reduction was more pronounced in vivo than in vitro experiments.

**Conclusion:**

High-polar constituents primarily mediated the antimutagenic activity of CEES and AFES. Meanwhile, other phytoconstituents in CEES, such as moderately polar and nonpolar constituents, synergistically potentiated the genoprotective activity, resulting in greater efficacy of CEES than AFES.

**Supplementary Information:**

The online version contains supplementary material available at 10.1186/s41021-025-00341-z.

## Introduction

Ethyl methanesulfonate (EMS, CH_3_SO_3_C_2_H_5_) is a volatile organic solvent with a colorless to faint yellow liquid. It is an ethyl ester of methanesulfonic acid, synthesized through the reaction of ethyl alcohol with either methanesulfonic anhydride (CH_3_SO_2_–O–SO_2_CH_3_) or methanesulfonyl chloride (CH_3_SO_2_–Cl). EMS is a monofunctional alkylating agent used extensively in DNA repair studies as a mutagenic, teratogenic, and carcinogenic agent [1]. The ethyl group of EMS reacts with nucleophilic sites on DNA, producing ethylated nucleotides at: (1) highly nucleophilic N-atoms, such as N7-guanine and N3-adenine; and (2) to a lesser extent, nucleophilic O-atoms, such as O6-guanine and O2- thymine [2, 3]. The alkylated N-atoms cause chromosomal aberration, whereas alkylated O-atoms cause substitution point mutation (GC→AT) by mispairing O6-ethylguanine with T during DNA replication [2]. In vivo experiments have reported that intraperitoneal injection with EMS induces chromosomal damage in rodents at single doses ranging from 100 to 400 mg/kg [4, 5]. Furthermore, in vitro experiments have shown that EMS induces comet tail formation at concentrations ranging from 250 to 350 µg/mL for a short period (1–4 h) in primary cultured mammalian cells [6–8].

The instability of DNA structure remarkably increases the risk of cancer development, which may be mitigated by bioactive plant-derived compounds. The discovery of phytoconstituents is a powerful tool in developing the pharmaceutical and therapeutic industries. The Asteraceae family, generally known as the aster, daisy, composite, or sunflower family, has been receiving research attention due to its long history of use in traditional medicine [9]. Asteraceae, formerly Compositae, is one of the leading flowering plant families, including around 1600 genera and 25,000 species. The *Echinops* genus within this family comprises over 130 species distributed across Eurasia, North Africa and East Africa. *Echinops spinosus* (ES), known as spiny globe thistle, is traditionally used in folk medicine for its anti-inflammatory, diuretic, and digestive characteristics [10, 11].

The *Echinops* genus has a distinctive phytochemical fingerprint characterized by: (1) two novel sesquiterpenoids, echinopine A and echinopine B, featuring unique carbon frameworks; (2) five quinolone and quinazoline alkaloids, including echinopsine, echinorine, echinopsidine, echinozolinone, and 7-hydroxyechinozolinone; (3) a unique lipid (ritroyne A); and (4) thiophenes as the primary constituents in *Echinops* roots [10, 12, 13]. Alongside its phytochemical fingerprint, the *Echinops* genus has common bioactive compounds such as proteins, lipids, sugars, sugar alcohols, organic acids, polyphenols, phenolic acids and flavonoids [10, 14]. Antimutagenic properties of individual phenolic acids found in *Echinops* species, such as chlorogenic acid, gallic acid, and rosmarinic acid, have been documented against fungal toxin 3-nitropropionic acid, cyclophosphamide, and doxorubicin, respectively [15–17]. However, synergistic interaction of the complex phytochemicals within the *E. spinosus* may further enhance its distinctive antimutagenic activity. Recent studies have demonstrated that three *Echinops* species (*E. spinosus*, *E. ritro*, and *E. cephalotes*) exhibit nephroprotective, hepatoprotective and cardioprotective activities in rats, respectively [18–20]. To our knowledge, this is the first study to examine the antimutagenic effects of *E. spinosus* using in vitro and in vivo genotoxic assays in mouse bone marrow and spleen. To identify the phytochemical nature responsible for genoprotective activity, we compared the efficacy of the crude extract of *E. spinosus* (CEES) with its aqueous fraction (AFES) to inhibit EMS-induced chromosomal and DNA damage.

## Materials and methods

### Extraction process

The aerial flowering parts of *E. spinosus* (700 g) were powdered and extracted using 70% ethanol, as described in our previous work [21]. Subsequently, CEES (64.17 g) was first suspended in hot water, left to stand overnight, and then filtered. The extract was partitioned successively with three solvents of different polarities: methylene chloride, *n-*butanol, and distilled water. The final aqueous residue was weighed, resulting in the isolation of AFES (41.82 g).

### Gas chromatography-mass spectrometry (GC–MS) analysis

The chemical constituents of CEES were identified as 73 primary metabolites, using GC**–**MS analysis, belonging to nitrogenous compounds, proteins, lipids, sugars, sugar alcohols, and organic acids, as described in our recent study [21]. The silylated derivatives of AFES were separated on an Rtx-5MS column (30 m × 0.25 mm), following the procedures described earlier [22]. The retention indices of chemical constituents of AFES were compared with those of standard n-alkanes (C6-C20, Sigma Aldrich Co, USA) using the AMDIS software (www.amdis.net). Further, their mass spectra were compared with data from the Wiley Spectral Library collection and NSIT Library database. Peak abundance data were exported for multivariate analyses using MET-IDEA software [23].

### Determination of the total phenolic content (TPC)

TPC was evaluated using the Folin-Ciocalteu reagent (FCR) colorimetric method, following the procedures described by Odumosu et al. [24] with minor modifications. An aliquot of 500 µL of the diluted sample in 50% methanol was mixed with 2.5 mL of FCR and 2.5 mL of sodium carbonate (75 g/L). The mixture reaction was vigorously vortexed and allowed to stand at room temperature for two hours. The absorbance was measured at 765 nm using a spectrophotometer, and the blank solution was prepared by substituting FCR with distilled water in the same reaction mixture. Gallic acid was used as a standard reference and was prepared under the same conditions. TPC was expressed as mg gallic acid equivalent per one gram of dried sample through the calibration curve of gallic acid.

### Determination of total flavonoid content (TFC)

TFC was measured using the AlCl_3_ colorimetric method as previously described in detail [24]. An aliquot of 200 µL of the diluted sample in 50% methanol was mixed with 75 µL of 5% NaNO_2_ for 5 min. Next, 1.25 mL of 10% AlCl_3_ and 0.5 mL of 1 M NaOH were added to the reaction mixture. The absorbance of the mixture was measured at 510 nm using a spectrophotometer, and the blank solution was prepared by replacing AlCl_3_ with distilled water in the same reaction mixture. Quercetin was used as a standard reference and was prepared under the same conditions to construct the calibration curve. TFC was expressed as mg quercetin equivalent per one gram of the dried extract through the calibration curve of quercetin.

### High performance liquid chromatography (HPLC) analysis

HPLC analysis of CEES and AFES was conducted using an Agilent 1260 series chromatograph. The separation was achieved with a Zorbax Eclipse plus C8 column (4.6 mm x 250 mm i.d., 5 μm). The mobile phase consisted of water (A) and 0.05% trifluoroacetic acid in acetonitrile (B), with a flow rate of 0.9 mL/min. The mobile phase was programmed to run a linear gradient as follows: at 0 min (82% A); 0–1 min (82% A); 1–11 min (75% A); 11–18 min (60% A); 18–22 min (82% A); 22–24 min (82% A). The multi-wavelength detector was monitored at 280 nm and 330 nm for phenolic and flavonoid constituents’ detection, respectively. The solution of the tested samples was injected in a volume of 5-µL, with the column maintained at 40 °C [25, 26]. The compounds were quantified by comparing their retention time and absorption spectra of all peaks of the sample with those of nineteen standard polyphenols. These standards included gallic acid, chlorogenic acid, catechin, methylgallate, coffeic acid, syringic acid, procatechol, rutin, ellagic acid, coumaric acid, vanillin, ferulic acid, naringenin, rosmarinic acid, daidzein, quercetin, cinnamic acid, kaempferol, and hesperetin, all of which were run under the same conditions. The concentration of the polyphenols in the sample was determined using the area response of each compound by interpolation in corresponding calibration graphs.

### Experimental animals

Adult male Swiss albino mice, aged 8–12 weeks old and weighing approximately 25 g, were obtained from the Animal House of the National Research Centre, Cairo, Egypt. The mice were housed in polycarbonate cages provided with wood sawdust bedding in a controlled room. This room was maintained under a light and dark cycle of 12-h each, at a temperature of 25 ± 2 °C, with humidity levels of 50 to 60%. The animals were supplemented with standard animal pellet chew and tap water. The experiments were conducted following National Institute of Health guidelines for the Care and Use of Laboratory Animals. The protocol was reviewed and approved by the Institutional Ethics Committee of the National Research Centre.

### Isolation of primary bone marrow and spleen cells

Mouse hind legs and spleens were excised under sterile conditions. Bone marrow was flushed out from the femur and tibia into a petri dish using a syringe filled with RPMI-1640 medium. The spleen was crushed using the plunger end of a syringe through a cell strainer (100 μm) over a petri dish containing RPMI-1640 medium. The cell suspension from each tissue was centrifuged at 1500 rpm for 10 min at room temperature. The resulting cell pellets were re-suspended in RPMI-1640 medium supplemented with 20% fetal bovine serum (FBS), antibiotics, and 5 µg/mL of concanavalin A Type IV (Con A; C 2010; Sigma-Aldrich Co., United States).

### In vitro cytotoxicity assay

Cytotoxic activities were assessed without mitogen concanavalin A addition, using colorimetric cell count kit-8 (CCK-8, Dojindo Laboratories, Kumamoto, Japan) as described previously [27]. Aliquots of 50 µL of each primary bone marrow and spleen cells (0.1 × 10^6^ cells/100 µL/well) were seeded into a 96-well plate for 24 h. Next, 50 µL of complete medium supplemented with five concentrations of CEES and AFES (75, 125, 250, 500, and 1000 µg/mL) was added to each well and incubated for 24 h. Then, CCK-8 (20 µL) was added per well, and the plate was incubated in a humidified incubator with 5% CO2 at 37ᵒC for an additional three hours. The absorbance of the microplate was measured at 450 nm using the ELISA microplate. The percentage of cell viability was measured by comparing the absorbance values of the control cultures with those of the treated cultures.

### In vitro primary cell treatment

Bone marrow and spleen cell suspensions (5 × 10^6^cells/mL) were cultured in Petri dishes for 24 h. After this incubation period, the cell cultures were treated as follows:

1. DMSO (1%) was used as a vehicle control and added to the cultures for 24 h.
2. CEES or AFES (500 µg/mL, dissolved in 1% DMSO) was incubated separately in the cell cultures for 24 h.
3. EMS (300 µg/mL; CAS-No: 62.50-0; Thermo Fisher Scientific Chemicals) was prepared freshly in distilled water and added to the cell cultures for two hours before the harvest.
4. Two concentrations of CEES and AFES (250 and 500 µg/mL) were added for 24 h, followed by EMS treatment (300 µg/mL) for two hours before the harvest.

The experiment was replicated three times for each concentration. Following treatment, half of the cell cultures were treated with colchicine two hours before the harvest period (48 h) for chromosomal analysis. The other half of the cultures were collected without colchicine treatment for DNA damage examination.

### In vivo acute toxicity (Median lethal dose, LD_50_)

The oral LD_50_ of CEES in male mice was greater than 5000 mg/kg, as reported in our work [21]. To determine the LD_50_ of AFES, six male mice were orally given AFES at a dose of 5000 mg/kg, dissolved in distilled water. The mice were observed for 24 h for any signs of toxicity or death [28].

### In vivo treatment schedule

Forty mice were equally allocated into eight groups (5 mice/group) as follows:

- Group 1: Control group received 0.1 mL of 10% DMSO via oral gavage for seven consecutive days.
- Groups (2–3): Mice were orally administered CEES, dissolved in 10% DMSO, and AFES, dissolved in distilled water, at a dose of 500 mg/kg for seven successive days. This dose was selected based on our recent study, which reported that the safety threshold dose of CEES in mice was ≤ 500 mg/kg following a 28-day repeated treatment [21].
- Group 4: Mice were intraperitoneally injected with a single dose of EMS (300 mg/kg diluted in distilled water) for 24 h.
- Groups (5–8): Mice were treated with CEES or AFES at doses of 250 and 500 mg/kg for seven days, followed by EMS one hour after the last dose of plant extract.

At 24 h after the end of the treatment period, the mice were authenized by cervical dislocation, and their hind legs were excised. Bone marrow cells were collected from femurs using phosphate buffer solution (PBS) for chromosomal preparation and from the tibias using FBS for MN examination.

### Chromosomal aberrations (CAs) assay

Chromosome preparation was prepared from cultured cell suspensions and mouse bone marrow, as previously described in detail [27]. The collected cells were centrifuged, and the cell pellets were incubated in a hypotonic solution (0.075 M KCl) at 37 °C for 20 min, followed by centrifugation at 1500 rpm for 10 min at room temperature. The cell pellets were fixed twice with a freshly prepared ice-cold fixative (3:1 methanol: glacial acetic acid). Subsequently, drops of cell suspension were placed onto clean microscopic slides. The smears were dried and stained with 10% Giemsa in PBS (0.06 M Na_2_HPO_4_ and 0.06 M KH_2_PO_4_, pH 6.8) for 10 min. Metaphases with structural CAs, such as gaps, breaks, fragments, and Robertosonian translocations (RT, or centric fusion), were examined using light microscopy at a magnification of ×1500. The analysis of well-spread metaphases was conducted as follows: (1) In vitro experiments scored 300 metaphases per concentration (100 metaphases for each experiment), and (2) in vivo experiments examined 500 metaphases per dose (100 metaphases for each mouse in the group).

### Comet assay (Single cell gel Electrophoresis, SCGE)

Standard alkaline SCGE assay was performed as described previously [29]. The cell suspension (100 µL) was mixed with low-melting agarose (1000 µL, 0.8%), and this mixture was spread over full frosted slides precoated with normal agarose (1.0%). The coverslips were placed on the slides, creating a thin layer of agarose and allowed to solidify at 4 °C for 5 min. The coverslips were carefully removed, and the slides were immersed in a lysis buffer (2.5 M NaCl, 100 mM Na_2_EDTA, 10 mM Tris HCl, pH 10, 1% Triton X-100, and 10% DMSO) for 2-h at 4◦C in the dark. Next, the slides were exposed to an alkaline buffer (300 mM NaOH, 1 mM Na_2_EDTA, pH >13.0) for 20 min to unwind DNA. Electrophoresis was then conducted at 25 V and 300 mA for 30 min. Finally, the slides were neutralized in 0.4 M Tris buffer (pH 7.5) for 5 min and then fixed in absolute ethanol. The air-dried slides were stained with ethidium bromide and examined using fluorescent microscopy equipped with a green excitation filter (510–560 nm) and a barrier filter (590 nm). Six hundred cells were analyzed per concentration (200 cells for each experiment) using automatic comet score™ software (TriTek Corp, version 2.0.0.0, Sumerduck, VA 22742, United States). The percentage of DNA in the comet tail (% tail DNA) and Olive tail moment (OTM) are quantitative and qualitative measures of fragmented DNA within the cells, respectively. The % tail DNA represents the proportion of fragmented DNA that migrated from the cell nucleus into the comet tail. In contrast, OTM describes heterogeneity within the cell population or varying degrees of DNA fragmentation in the comet tail.

### Micronucleus (MN) assay

Bone marrow cells were collected in fetal bovine serum, smeared onto slides, air-dried, and then fixed in absolute methanol. The slides were stained using the May-Grünwald Giemsa protocol and examined under light microscopy at a magnification of ×1500 [30]. Ten thousand polychromatic erythrocytes (PCEs) were analyzed per dose (2,000 PCEs per mouse) to determine the presence of micronucleated polychromatic erythrocytes (MNPCEs). MN appears as a small, round or oval-shaped body, and its size ranges from 1/5 to 1/20 the diameter of PCE.

### Data analysis

The data were processed using Statistical Package for the Social Sciences (SPSS, version 26, IBM Corp, New York). The differences in means among the experimental groups were analyzed using a one-way analysis of variance (ANOVA), followed by Duncan’s multiple-range test. The results were considered statistically significant when the *P*-value was < 0.05.

The reduction in mutagenicity was calculated as follows:

$$\begin{aligned} \mathrm{Reduction}\;\mathrm{index}\;(\mathrm{RI}) =& [\mathrm{positive}\;\mathrm{group} - \textrm{co-treated}\;\mathrm{group}]/ \\&[\mathrm{positive}\;\mathrm{group} - \mathrm{control}\;\mathrm{group})\end{aligned}$$

## Results

### Phytochemical analysis

In Table 1, GC-MS analysis identified 25 compounds in AFES, representing various chemical classes. Nitrogenous compounds and amino acids were dominant (77.13% of total silylated metabolites), with bis-n-(trimethylsilyl) ethylamine as the primary constituent (50.05%). Nine compounds were found at a minor concentration of < 1%. As shown in Tables 2 and 3, CEES remarkably demonstrated higher TPC (3.5-fold increase) and TFC (3.1-fold increase) than AFES. HPLC analysis identified 17 and 14 compounds in CEES and AFES, respectively. In CEES, chlorogenic acid, rosmarinic acid, ferulic acid, and gallic acid were the main constituents, which accounted for 42.3%, 17.1%, 15.1%, and 7.6% of total compounds, respectively. In AFES, chlorogenic acid and gallic acid were dominant constituents and represented 58.8% and 31.1% of total compounds, respectively.

**Table 1:** GC-MS analysis of silylated metabolites in AFES

No.	Compound name	Formula	Class	RT	AFES (%)
1	2-Aminobutyric acid, bis-TMS	C_10_H_25_NO_2_Si_2_	AA	3.68	1.96
2	Diethylformamide	C_5_H_11_NO	NC	3.81	3.27
3	Suberic acid-diTMS	C_14_H_30_O_4_Si_2_	FA	4.03	0.84
4	N-Ethyl, N-vinylacetamide	C_6_H_11_NO	NC	4.1	5.73
5	Glycine, N-propanoyl, TMS	C_8_H_17_NO_3_Si	AA	4.15	3.65
6	2-Octene, 3,7-dimethyl-, (Z)-	C_10_H_2_0	UH	4.42	5.02
7	Ethylamine, bis-n-(trimethylsilyl)-	C_8_H_23_NSi_2_	NC	4.59	50.45
8	Acetamide, N,N-diethyl-	C_6_H_13_NO	NC	4.68	3.86
9	Di-N-propyl methylamine	C_7_H_17_N	NC	4.83	3.54
10	2-Propanamine, N-ethyl-	C_5_H_13_N	NC	5.03	6.62
11	Pyruvic acid MEOX TMS	C_7_H_15_NO_3_Si	OA	6.14	0.75
12	Tris(trimethylsilyl)carbamate	C_10_H_27_NO_2_Si_3_	NC	6.26	0.63
13	D-Lactic acid-diTMS	C_9_H_22_O_3_Si_2_	OA	6.45	1.07
14	Carbonic acid-diTMS	C_7_H_18_O_3_Si_2_	OA	7.78	1.05
15	Glyoxalic hydrate-triTMS	C_11_H_28_O_4_Si_3_	OA	8.35	0.49
16	Phosphoric acid, TMS	C_9_H_27_O_4_PSi_3_	OC	11.51	2.25
17	γ-Aminobutyric acid, tri-TMS	C_13_H_33_NO_2_Si_3_	AA	12.41	1.32
18	2-Aminoethanol-triTMS	C_11_H_31_NOSi_3_	AA	14.18	0.66
19	Epitestosterone, TMS	C_22_H_36_O_2_Si	ST	16.35	1.11
20	L-(-)-Arabitol, 5TMS	C_20_H_52_O_5_Si_5_	SAL	21.18	0.51
21	Palmitic acid, TMS	C_19_H_40_O_2_Si	FA	25.78	1.65
22	Linoleic acid trimethylsilyl esterI	C_21_H_40_O_2_Si	FA	28.22	0.77
23	.(E)−9-Octadecenoic acid, TMS ester	C_21_H_42_O_2_Si	FA	28.34	1.28
24	Stearic acid-monoTMS	C_21_H_44_O_2_Si	FA	28.78	0.65
25	D-Turanose 7TMS	C_33_H_78_O_11_Si7	SU	35.31	0.68
Total					99.81

The Chemical constituents of CEES were reported previously in supplementary table S1 (21)

For easier readability, a comparison between CEES and AFES was provided in Supplementary file

*RT* Retention Time, *NC* Nitrogenous Compound, *UH* Unsaturated hydrocarbon, *AA* Amino Acid, *OC* Organosilicon Compound, *FA* Fatty Acid, *OA* Organic Acid, *SAL* Sugar Alcohol, *ST* Steroid, *SU* Sugar

**Table 2:** Phytochemical colormetric content in CEES and AFES

Variable	CFES	AFES
TPC(mg gallic acid equivalent/g extract)	200.15 ± 0.89^b^	57.73 ± 0.43^a^
TFC (mg quercetin equivalent/g extract)	164.36 ± 0.27^b^	53.40 ± 1.39^a^

Data were expressed as mean ± SE

Values within the same raw followed by different superscript letters are significantly different from another as calculated by One-way ANOVA followed by Duncan’s multiple range test

**Table 3:** HPLC analysis of CEES and AFES

No.	Constituents	Class	CEES		AFES	
			Area	Conc. (µg/g)	Area	Conc. (µg/g)
1	Gallic acid	Phenolic acid	644.36	2358.93	672.54	2974.40
2	Chlorogenic acid	Phenolic acid	1889.66	13165.95	867.51	5628.29
3	Catechin	Flavanol	-------	-------	-------	-------
4	Methyl gallate	Phenolic acid derivative	19.39	54.26	-------	-------
5	Coffeic acid	Phenolic acid	439.35	1127.01	85.54	330.97
6	Syringic acid	Phenolic acid	184.89	543.75	81.31	297.34
7	Pyrocatechol	Phenol	-------	-------	-------	-------
8	Rutin	Favonol Glycoside	11.01	82.44	-------	-------
9	Ellagic acid	Polyphenol	22.43	113.94	8.82	44.07
10	Coumaric acid	Phenolic acid	129.22	232.21	36.75	65.39
11	Vanillin	Phenolic aldehyde	430.83	781.30	4.96	9.22
12	Ferulic acid	Phenolic acid	1618.18	4696.77	5.30	15.41
13	Naringenin	Flavanone	41.30	190.59	14.85	67.85
14	Rosmarinic acid	Polyphenol	1094.40	5314.40	9.88	52.97
15	Daidzein	Isoflavone	63.95	183.07	1.48	4.16
16	Quercetin	Flavonol	19.85	123.64	-------	-------
17	Cinnamic acid	Phenolic acid	289.03	280.09	15.04	13.46
18	Kaempferol	Flavonol	366.22	460.33	9.01	28.43
19	Hesperetin	Flavanone	612.48	1434.00	18.84	46.31
Total				311142.68	9578.27	

### In vitro cytotoxicity of CEES and AFES

**Fig. 1:**
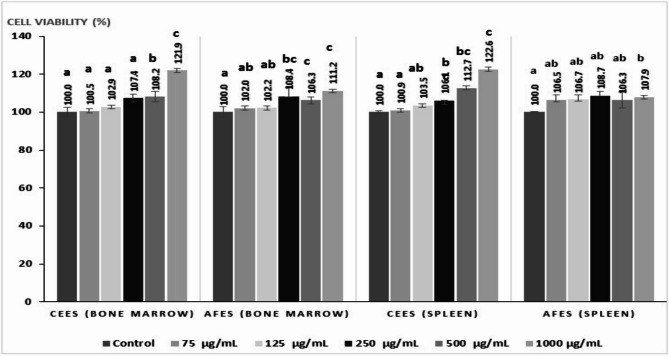
Effect of CEES and AFES on cell viability in non-stimulated mouse bone marrow and splenic cultures (i.e., cells in the resting stage of mitosis without concanavalin A stimulation) using CCK-8 assay Data expressed as mean ± S.E. Values with different superscript letters in bars are statistically different from one another as calculated by One-way ANOVA followed by Duncan’s multiple range test

CCK-8 findings showed CEES and AFES did not reduce the cell viability in primary mouse bone marrow and spleen cultures after 24 h of exposure at concentrations up to 1000 µg/mL. However, both treatments, particularly at 1000 µg/mL, increased the cell viability of both bone marrow and spleen cultures (Fig. 1).

### In vitro genoprotective effect of CEES and AFES

In Table 4, treatment with CEES or AFES alone (500 µg/mL, 24 h) showed no statistical impact on the CAs levels in the bone marrow and splenic cultures compared to their control values. However, EMS treatment (300 µg/mL, 2-h) induced a marked increase in CAs levels in bone marrow (19.33%, i.e., a 7.2-fold increase) and splenic (16.33%, i.e., 4.9-fold increase) cultures compared to their control values. Metaphases with breaks and fragments were predominant in EMS-treated cultures. Pretreatment with high concentrations of CEES and AFES (500 µg/mL) significantly attenuated EMS-induced chromosomal damage, with reduction indices of 34.00% in bone marrow and 46.15% in spleen cultures. In contrast, treatment with CEES and AFES at a low concentration (250 µg/mL) resulted in marginal reductions in CAs compared to the EMS-treated cultures.

**Table 4:** In vitro genoprotective activity of CEES and AFES against chromosomal damage induced by EMS in mouse bone marrow and splenic cultures

Treatments	No. of different types of abnormal metaphase of CAs												
	Bone marrow						Spleen						
	Gap	Fr/Br	MA	Total	Mean% ± S.E	*R*.I (%)	Gap	Fr/Br	MA	Total	Mean%± S.E		*R*.I (%)
Control	4	4	0	8	2.67 ± 0.67^a^	-	5	5	0	10	3.33 ± 0.33^a^		
CEES (LC)	3	9	0	12	4.00 ± 0.58^a^	-	6	7	0	13	4.33 ± 0.67^a^		
AFES (HC)	2	11	0	13	4.33 ± 0.88^a^	-	4	10	0	14	4.67 ± 0.88^a^		
EMS	2	35	21	58	19.33 ± 1.76^d^	-	4	32	13	49	16.33 ± 2.03^d^		
EMS + CEES (LC)	5	30	14	49	16.33 ± 0.67^cd^	18.00	5	19	16	40	13.60 ± 1.20^cd^	23.08	
EMS + CEES (HC)	4	21	16	41	13.67 ± 0.88^b^	34.00	3	22	6	31	10.33 ± 0.67^b^	46.15	
EMS + AFES (LC)	8	27	16	51	17.00 ± 1.15^cd^	14.00	4	27	10	41	13.67 ± 0.88^cd^	20.51	
EMS + AFES (HC)	2	26	19	47	15.67 ± 0.88^bc^	22.00	2	24	12	38	12.67 ± 0.67^bc^	28.21	

LC: Low concentration (250 µg/mL); HC: High concentration (500 µg/mL)

Three hundred metaphases were analyzed per treatment (three cultures/treatment; 100 metaphases/culture)

Fr/Br: Fragment and/or Break; MA: Multiple aberrations; R.I: Reduction index

The values with different superscript letters in each column are significantly different from one another as calculated by One-way ANOVA followed by Duncan’s multiple range test

In Table 5, treatment with the high concentration of CEES or AFES did not induce substantial increases in the comet tail parameters in mouse bone marrow and spleen cultures. By contrast, EMS treatment increased the OTM value and percentage tail DNA in bone marrow (10.93%, representing a 2.9-fold increase) and spleen (7.49%, representing a1.72-fold increase) cultures. A 24-hour pretreatment with CEES or AFES significantly reduced EMS-induced comet tail formation compared to EMS-treated cultures. CEES (500 µg/mL) was more effective in restoring the percentage tail DNA in bone marrow (4.71%, representing a 2.3-fold decrease) and spleen (4.57%, representing a 1.6-fold decrease) to the control level.

**Table 5:** In vitro genoprotective activity of CEES and AFES against DNA comet tail formation induced by EMS in mouse bone marrow and splenic cultures

Treatment	Bone marrow		Spleen	
	% Tail DNA	OTM (A.U)	% Tail DNA	OTM (A.U)
Control	3.67 ± 0.07^ab^	1.03 ± 0.02^a^	4.34 ± 0.09^a^	1.08 ± 0.009^a^
CEES (LC)	3.21 ± 0.05^a^	1.10 ± 0.02^a^	4.44 ± 0.09^a^	1.26 ± 0.01^ab^
AFES (HC)	3.59 ± 0.09^ab^	1.10 ± 0.03^a^	4.13 ± 0.15^a^	1.15 ± 0.03^a^
EMS	10.93 ± 0.89^e^	3.12 ± 0.27^d^	7.49 ± 0.49^c^	2.21 ± 0.23^d^
EMS + CEES (LC)	5.39 ± 0.15^bc^	1.61 ± 0.10^b^	5.84 ± 0.18^b^	1.48 ± 0.03^bc^
EMS + CEES (HC)	4.71 ± 0.16^b^	1.50 ± 0.06^b^	4.57 ± 0.12^a^	1.65 ± 0.04^c^
EMS + AFES (LC)	7.23 ± 0.57^d^	1.98 ± 0.09^c^	5.74 ± 0.42^b^	1.57 ± 0.03^c^
EMS + AFES (HC)	6.71 ± 0.37^d^	1.82 ± 0.07^bc^	5.61 ± 0.36^b^	1.58 ± 0.06^c^

Data were expressed as Mean ± S.E; OTM is expressed in arbitrary unit (A.U)

LC: Low concentration (250 µg/mL); HC: High concentration (500 µg/mL)

Six hundred cells were analyzed per treatment (three cultures/treatment; 200 cells/culture). The values with different superscript letters in each column are statistically significantly different from one another as calculated by One-way ANOVA followed by Duncan’s multiple range test

### In vivo preventive effect of CEES and AFES

**Fig. 2:**
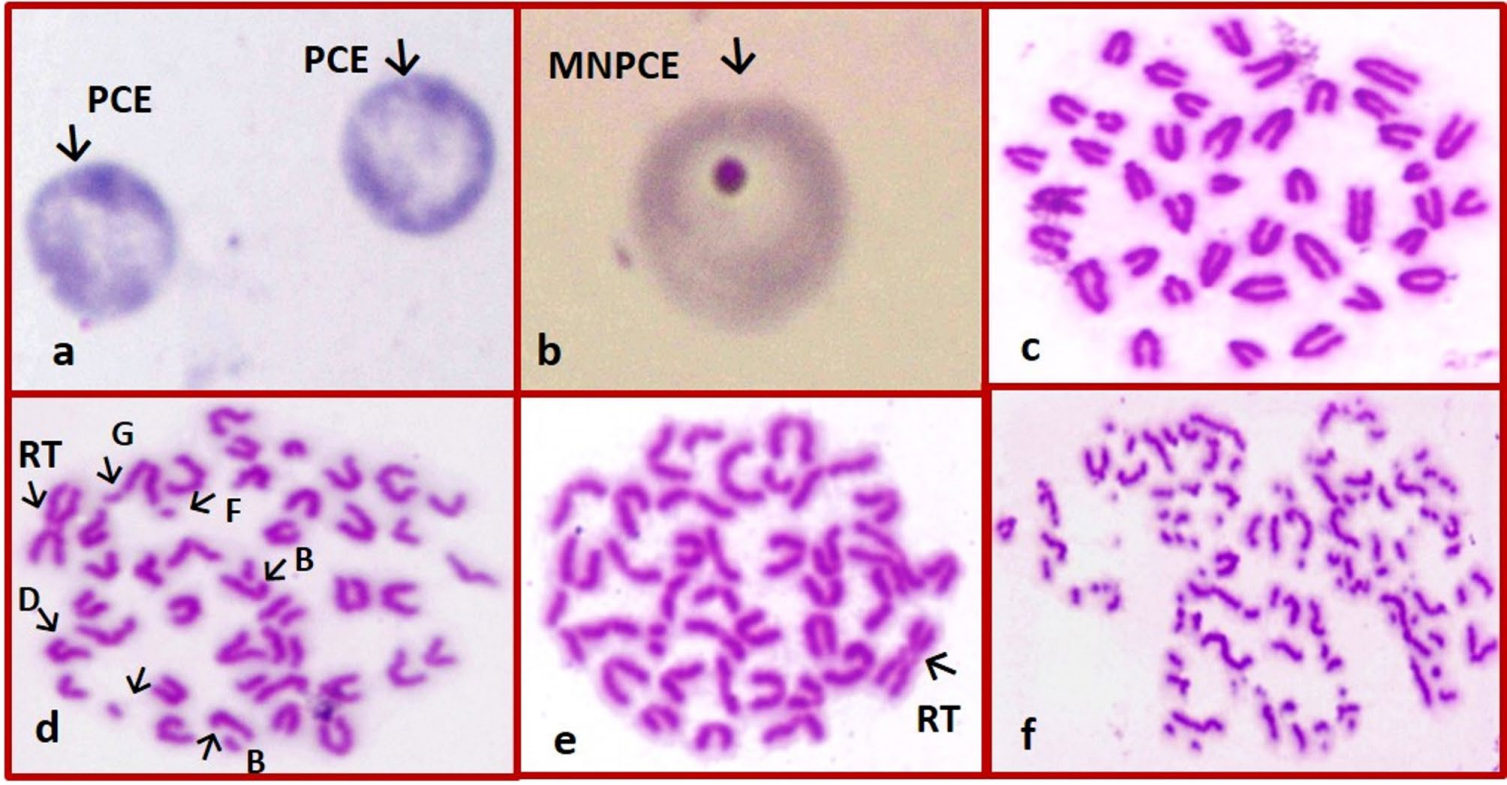
Photomicrographs of chromosomal damage in mouse bone marrow cells showing: **a** PCEs; **b** MNPCE; **c** control metaphase; **d** metaphases with gap (G), break (B), fragment (F), deletion (D), and Robertsonian translocation (RT); **e** metaphase with RT; **f** metaphase with fragmentation

No signs of death or toxicity were reported in mice treated with a single oral dose of AFES at 5000 mg/kg during the initial 24 h observation period or the 14-day follow-up. In Table 6, individual treatment with CEES or AFES did not affect CAs (4.40% and 4.80%, respectively) and MNPCEs (1.04% and 1.14%, respectively) compared to their control values. By contrast, EMS significantly increased CAs (27.0%, representing a 7.5-fold increase) and MNPCEs (14.14%, representing a 16.8-fold increase) in mouse bone marrow cells compared to the control group. Breaks or fragments and RT were predominant types of CAs observed in the EMS-treated group (Fig. 2). These genetic damages considerably decreased when CEES and AFES were given before EMS administration. The improvements in CAs and MNPCs were more pronounced in the groups that received the high dose of CEES (54% and 56%, respectively) and AFES (50% and 49%, respectively).

**Table 6:** In vivo genoprotective activity of CEES and AFES against EMS-induced chromosomal damage in mouse bone marrow cells

Groups	No. of different types of abnormal metaphase of CAs							MNPCEs		
	Gap	Fr/Br	MA	RT	Total	Mean% ± S.E	*R*.I (%)	No.	Mean% ± S.E	*R*.I (%)
Control	7	11	-	-	18	3.60 ± 0.51^a^	-	84	0.84 ± 0.12^a^	-
CEES (HD)	7	15	-	-	22	4.40 ± 0.60 ^a^	-	104	1.04 ± 0.15^a^	-
AFES (HD)	6	18	-	-	24	4.80 ± 0.58 ^a^	-	114	1.14 ± 0.21^a^	-
EMS	6	58	49	22	135	27.00 ± 0.89 ^e^	-	1414	14.14 ± 0.65^d^	-
EMS + CEES (LD)	10	52	18	10	90	18.00 ± 0.55 ^cd^	38	858	8.58 ± 0.35^c^	42
EMS + CEES (HD)	3	42	21	6	72	14.40 ± 0.93 ^b^	54	672	6.72 ± 0.48^b^	56
EMS + AFES (LD)	9	44	40	11	104	20.80 ± 1.30 ^d^	26	876	8.76 ± 0.56^c^	40
EMS + AFES (HD)	6	43	19	8	76	15.20 ± 1.20 ^bc^	50	768	7.68 ± 0.37^bc^	49

LD: Low dose (250 mg /mL); HD: High dose (500 mg /mL)

Five hundred metaphases were analyzed per group (5 mice / group; 100 metaphases / mouse)

Fr/Br: Fragment and/or Break; MA: Multiple aberrations; R.I: Reduction index

Total 10,000 PCEs were scored per group (5 mice / group; 2,000 PCEs/ mouse)

The values with different superscript letters in each column are statistically significantly different from one another as calculated by One-way ANOVA followed by Duncan's multiple range test

## Discussion

In this study, AFES contained a higher concentration of nitrogenous compound bis-n-(trimethylsilyl ethylamine (50.01%, i.e., a 2.9 fold increase) compared to the CEES (17.01%) as identified by GC-MS analysis [21]. On the other hand, CEES had higher TPC and TFC than AFES, which can be due to the presence of 17 and 14 compounds identified in each sample, respectively, using HPLC analysis. Surprisingly, AFES had higher concentrations of chlorogenic acid (58.76%) and gallic acid (31.05%) compared to those in CEES (42.28% and 7.57%, respectively). These results highlight that CEES efficiently extracted a wide range of phytochemical constituents, which are soluble selectively in different solvents. Water is a polar solvent that selectively concentrates hydrophilic nitrogenous and phenolic compounds, thus enriching their concentrations in AFES.

 In vitro cytotoxic experiments showed that CEES and AFES exhibited non-toxic effects on bone marrow and splenic cultures, at concentrations up to 1000 µg/mL. Surprisingly, high concentrations of both treatments (≥ 500 µg/mL) caused an increase in the cell viability, indicating the proliferative or mitogenic activity of phytochemicals presented in ES. Consistent with this potential, ES roots enhance the wound-healing process in rat skin tissues by stimulating immune cell proliferation and displaying astringent, antimicrobial, antioxidant, and anti-inflammatory properties [31]. In acute oral toxicity, AFES exhibited an LD_5__0_ >5000 mg/kg in male mice, consistent with *Echinops* species (*spinosus*, *kebericho*, and *echinatus*), where LD_50_ values ranged from 2000 to 5000 mg/kg in rodents [21, 32, 33].

 In vitro experiments demonstrated that EMS substantially induced CAs and comet tail formation in mouse bone marrow and splenic cultures. Further, EMS caused in vivo chromosomal damage in mouse bone marrow, as indicated by a significant increase in CAs and MNPCEs. Notably, the most frequent types of CAs observed were breaks and RTs, which are hallmarks of clastogenicity and mutagenicity, respectively. However, the absence of RTs in “*in vitro”* experiments can be due to two main reasons: (1) the predominance of different types of CAs (e.g., gap, break, or fragment); (2) the requirement for higher concentrations or long-term exposure over two hours to detect this type of aberration. These findings indicate that EMS attacks the nucleophilic center of DNA, producing various DNA lesions, such as DNA adducts, cross-links, and strand breaks. These lesions are manifested cytogenetically as CAs and MNPCEs. Literature reviews demonstrated that EMS induces point mutation, MNPCEs, CAs, and comet tail formation in many rodent somatic cells [5, 34–36].

 In vitro experiments clarified that bone marrow cells were more susceptible to EMS-induced genotoxicity than spleen cells. However, splenic cultures exhibited higher responsiveness to the DNA-protective activity of CEES and AFES than bone marrow cells. These data suggest that a genotoxic or antigenotoxic response is likely associated with tissue configuration variation, tissue-specific repair efficiency, and the proliferation rate in each tissue, which contribute to the complete expression or fixation of mutations [37]. In this regard, Suzuki et al. [4] reported that EMS treatment (400 mg/kg, i.p injection) induces higher mutations in bone marrow cells with high mitotic rates (5-fold increase) than in liver cells with slow mitotic rates (>2-fold increase) in *lacZ* transgenic mice.

In both in vitro and in vivo experiments, individual treatment with CEES and AFES displayed no genotoxic effects in mouse bone marrow and spleen. Furthermore, supplementation with CEES and AFES decreased both chromosomal and DNA damage induced by EMS, and CEES was more effective than AFES, particularly at the high dose. These findings suggest that the antimutagenic activities of CEES and AFES result from highly polar compounds in both treatments. The superior antimutagenic effect of CEES is due to the synergistic interaction among its phytochemicals, both polar and nonpolar constituents, which enhances the bioavailability of polar constituents, thereby improving the overall genoprotective effects of CEES. Notably, the observed antimutagenic activity of AFES at the high dose can be due to highly polar constituents, which need a sufficient dose to exert their antimutagenic effects. In contrast, CEES provides additional benefits because of its broader phytochemical profile.

In this study, CEES and AFES exhibited higher inhibitory activity against EMS-induced chromosomal damage in vivo than in vitro*.* This observation indicates that in vivo genoprotective activity can be enhanced through two main factors: (1) metabolic activation of phytochemicals; and (2) prolonged exposure to these phytochemicals, allowing for cumulative protective effects [38]. In contrast, cultured cells treated with phytochemicals for limited hours lacked hormonal or growth factors, which might be necessary for optimal protection. In cultured splenic cells treated with a high concentration of CEES and EMS, the percentage of tail DNA reached the control level; however, the decrease in OTM and CAs persisted above control values. This data indicates that CEES effectively reduced total initial breaks (% tail DNA) but not all complex DNA damages (OTM), which distorted DNA supercoiling and affected DNA migration, causing a residual level of CAs.

These results appear novel, as comparable studies have not demonstrated similar findings involving any *Echinops* species. The antimutagenic activity of *Echinops* species may result from the synergistic interaction of its phytoconstituents, including organic acids, amino acids, fatty acids, sugars, sugar alcohols, phenolic acids, flavonoids, quinoline alkaloids, thiophenes, sesquiterpenoids, and sterols [10]. These phytochemicals likely act as desmutagens through several mechanisms: (1) direct interaction with DNA to prevent alkylation and stabilize genomic integrity; (2) activation of DNA repair mechanisms; and (3) modulation of cell cycle checkpoints [39].

## Conclusion

This study provides novel information about the antimutagenic activity of ES against EMS-induced chromosomal and DNA damage in mice. Treatment with CEES displayed a greater genoprotective effect compared to AFES. The superior efficacy of CEES is likely attributable to synergistic interactions between its high polar constituents and other bioactive compounds in AFES. However, this study has several limitations: (1) lack of comparison of the antimutagenic activities of other organic fractions of ES prohibits identification the most effective fraction and isolation the primary protective phytoconstituents; and (2) The underlying protective mechanisms of ES remain unclear and need further study to explore potential protection pathways, such as antioxidant effects, and activation of DNA repair pathway.

## Supplementary Information

Supplementary Material 1.

## Data Availability

All data generated or analyzed during this study are included in this published article.
