# Supplementary material for: In vitro and in vivo antimutagenic activity of Echinops spinosus crude extract and its aqueous fraction in mouse bone marrow and spleen
Source: Genes Environ. 2025 Nov 5;47:21. doi: 10.1186/s41021-025-00341-z (PMC12587627; doi:10.1186/s41021-025-00341-z)
Supplement: Supplementary file 1 — Supplementary Material 1. [file 41021_2025_341_MOESM1_ESM.docx]

**Comparative summary of the chemical constituents of CEES* and AFES using GC-MS analysis**

***The Chemical constituents of CEES were reported previously (21), DOI (10.1016/j.mrgentox.2025.503854)**

| Peak No. | Compound name | formula | Class | RT | CEES* (%) | AFES (%) |
| --- | --- | --- | --- | --- | --- | --- |
| 1 | 2-Aminobutyric acid, bis-TMS | C_10_H_25_NO_2_Si_2_ | AA | 3.68 | 1.05 | 1.96 |
| 2 | Diethylformamide | C_5_H_11_NO | NC | 3.81 | ------- | 3.27 |
| 3 | Suberic acid-diTMS | C_14_H_30_O_4_Si_2_ | FA | 4.03 | 0.36 | 0.84 |
| 4 | N-Ethyl,N-vinylacetamide | C_6_H_11_NO | NC | 4.1 | 2.75 | 5.73 |
| 5 | Glycine, N-propanoyl, TMS | C_8_H_17_NO_3_Si | AA | 4.15 | ------- | 3.65 |
| 6 | 2-Octene, 3,7-dimethyl-, (Z)- | C_10_H_2_0 | UH | 4.42 | 1.51 | 5.02 |
| 7 | Ethylamine, bis-n-(trimethylsilyl)- | C_8_H_23_NSi_2_ | NC | 4.59 | 17.05 | 50.45 |
| 8 | Acetamide, N,N-diethyl- | C_6_H_13_NO | NC | 4.68 | 1.22 | 3.86 |
| 9 | Di-N-propyl methylamine | C_7_H_17_N | NC | 4.83 | 1.34 | 3.54 |
| 10 | 2-Propanamine, N-ethyl- | C_5_H_13_N | NC | 5.03 | 2.59 | 6.62 |
| 11 | Pyruvic acid MEOX TMS | C_7_H_15_NO_3_Si | OA | 6.14 | 0.51 | 0.75 |
| 12 | Tris(trimethylsilyl)carbamate | C_10_H_27_NO_2_Si_3_ | NC | 6.26 | 0.22 | 0.63 |
| 13 | D-Lactic acid-diTMS | C_9_H_22_O_3_Si_2_ | OA | 6.45 | 0.52 | 1.07 |
| 14 | Glycolic acid, (2TMS) | C_8_H_20_O_3_Si_2_ | OA | 6.67 | 0.27 | ------- |
| 15 | Alanine-diTMS | C_9_H_23_NO_2_Si_2_ | AA | 7.38 | 0.29 | ------- |
| 16 | Carbonic acid-diTMS | C_7_H_18_O_3_Si_2_ | OA | 7.78 | 0.37 | 1.05 |
| 17 | Glyoxalic hydrate-triTMS | C_11_H_28_O_4_Si_3_ | OA | 8.35 | 0.16 | 0.49 |
| 18 | Malonic acid 2TMS | C_9_H_20_O_4_Si_2_ | OA | 9.38 | 0.35 | ------- |
| 19 | Urea, N,N'-bis(trimethylsilyl)- | C_7_H_20_N_2_OSi_2_ | NC | 9.90 | 0.31 | ------- |
| 20 | Glycerol-triTMS | C_12_H_32_O_3_Si_3_ | AL | 11.2 | 0.23 | ------- |
| 21 | Phosphoric acid, TMS | C_9_H_27_O_4_PSi_3_ | OC | 11.51 | 2.52 | 2.25 |
| 22 | Proline 2TMS | C_11_H_25_NO_2_Si_2_ | AA | 11.68 | 1.82 | ------- |
| 23 | Succinic acid (2TMS) | C_10_H_22_O_4_Si_2_ | OA | 11.84 | 0.28 | ------- |
| 24 | γ-Aminobutyric acid, tri-TMS | C_13_H_33_NO_2_Si_3_ | AA | 12.41 | 0.57 | 1.32 |
| 25 | Glyceric acid, TMS | C_12_H_30_O_4_Si_3_ | OA | 12.62 | 0.21 | ------- |
| 26 | Pipecolinic acid, (2TMS) | C_12_H_27_NO_2_Si_2_ | AA | 13.20 | 0.53 | ------- |
| 27 | Threonine 3TMS | C_13_H_33_NO_3_Si_3_ | AA | 13.97 | 0.21 | ------- |
| 28 | 2-Aminoethanol-triTMS | C_11_H_31_NOSi_3_ | AA | 14.18 | 0.28 | 0.66 |
| 29 | Malic acid TMS | C_13_H_30_O_5_Si_3_ | OA | 15.96 | 3.60 | ------- |
| 30 | Pyroglutamic acid, bis(trimethylsilyl)- | C_11_H_23_NO_3_Si_2_ | AA | 16.14 | 0.24 | ------- |
| 31 | Epitestosterone, TMS | C_22_H_36_O_2_Si | ST | 16.35 | 0.49 | 1.11 |
| 32 | Erythrose-triTMS | C_13_H_32_O_4_Si_3_ | SAL | 16.61 | 0.40 | ------- |
| 33 | Erythritol per-TMS | C_16_H_42_O_4_Si_4_ | SAL | 16.86 | 0.84 | ------- |
| 34 | Deoxy-erythro-pentonic acid, tetrakis-TMS | C_17_H_42_O_5_Si_4_ | SU | 18.33 | 0.21 | ------- |
| 35 | Asparagine 3TMS | C_13_H_32_N_2_O_3_Si_3_ | AA | 19.39 | 0.45 | ------- |
| 36 | Ribitol, 5TMS | C_20_H_52_O_5_Si_5_ | SAL | 19.59 | 0.34 | ------- |
| 37 | Vanilethanediol 3TMS | C_18_H_36_O_4_Si_3_ | OA | 20.91 | 0.52 | ------- |
| 38 | L-(-)-Arabitol, 5TMS | C_20_H_52_O_5_Si_5_ | SAL | 21.18 | 5.20 | 0.51 |
| 39 | Azelaic acid, bis-TMS | C_15_H_32_O_4_Si_2_ | FA | 21.51 | 0.85 | ------- |
| 40 | D-(-)-Tagatofuranose, 6TMS (isomer 2) | C_21_H_52_O_6_Si_5_ | SU | 21.88 | 0.94 | ------- |
| 41 | Glucofuranose 5tms | C_21_H_52_O_6_Si_5_ | SU | 22.18 | 0.55 | ------- |
| 42 | α-D-galactoside, methyl tetrakis-O-(trimethylsilyl)- | C_19_H_46_O_6_Si_4_ | SU | 22.29 | 0.29 | ------- |
| 43 | Citric acid-tetraTMS | C_18_H_40_O_7_Si_4_ | OA | 22.45 | 0.58 | ------- |
| 44 | Myristic acid-monoTMS | C_17_H_36_O_2_Si | FA | 22.50 | 0.19 | ------- |
| 45 | D-(-)-Fructofuranose, pentakis(trimethylsilyl) ether (isomer 1) | C_21_H_52_O_6_Si_5_ | SU | 22.66 | 1.53 | ------- |
| 46 | D-Psicofuranose, pentakis(trimethylsilyl) ether (isomer 1) | C_21_H_52_O_6_Si_5_ | SU | 22.78 | 1.36 | ------- |
| 47 | D-(-)-Fructopyranose, pentakis(trimethylsilyl) ether (isomer 1) | C_21_H_52_O_6_Si_5_ | SU | 22.94 | 1.40 | ------- |
| 48 | A-mannofuranoside-1-methyl-2,3,5,6-tetraTMS | C_19_H_46_O_6_Si_4_ | SU | 23.14 | 0.47 | ------- |
| 49 | Quinic acid-pentaTMS | C_22_H_52_O_6_Si_5_ | OA | 23.56 | 1.93 | ------- |
| 50 | D-Allofuranose, pentakis(trimethylsilyl) ether | C_21_H_52_O_6_Si_5_ | SU | 23.82 | 0.47 | ------- |
| 51 | L-(-)-Sorbose, pentakis(trimethylsilyl) ether | C_21_H_52_O_6_Si_5_ | SU | 23.87 | 0.40 | ------- |
| 52 | α-D-galactoside, methyl tetrakis-O-(trimethylsilyl | C_19_H_46_O_6_Si_4_ | SU | 24.05 | 1.43 | ------- |
| 53 | Ribitol, 1,2,3,4,5-pentakis-O-(trimethylsilyl)- | C_20_H_52_O_5_Si_5_ | SAL | 24.45 | 0.29 | ------- |
| 54 | 1,5-Anhydro-D-sorbitol, tetrakis(trimethylsilyl) ether | C_18_H_44_O_5_Si_4_ | SAL | 24.73 | 1.61 | ------- |
| 55 | D-Mannitol, 1,2,3,4,5,6-hexakis-O-(trimethylsilyl)- | C_24_H_62_O_6_Si_6_ | SAL | 25.02 | 1.64 | ------- |
| 56 | Myo-Inositol, 1,2,3,4,5,6-hexakis-O-(trimethylsilyl)- | C_24_H_60_O_6_Si_6_ | SAL | 25.16 | 0.45 | ------- |
| 57 | myo-Inositol (6TMS) | C_24_H_60_O_6_Si_6_ | SAL | 25.40 | 0.85 | ------- |
| 58 | β-D-Glucopyranose, TMS | C_21_H_52_O_6_Si_5_ | SU | 25.60 | 0.60 | ------- |
| 59 | D-Allofuranose, 5 TMS | C_21_H_52_O_6_Si_5_ | SU | 25.67 | 0.59 | ------- |
| 60 | Palmitic acid, TMS | C_19_H_40_O_2_Si | FA | 25.78 | 3.99 | 1.65 |
| 61 | Galactonic acid hexa-TMS | C_24_H_60_O_7_Si_6_ | SA | 26.06 | 0.90 | ------- |
| 62 | Linoleic acid, methyl ester | C_19_H_34_O_2_ | FA | 26.26 | 0.63 | ------- |
| 63 | Methyl isostearate | C_19_H_38_O_2_ | FA | 26.92 | 0.37 | ------- |
| 64 | Caffeic acid (3TMS) | C_18_H_32_O_4_Si_3_ | OA | 27.30 | 0.65 | ------- |
| 65 | D-Pinitol, pentakis(trimethylsilyl) ether | C_22_H_54_O_6_Si_5_ | SAL | 27.49 | 1.56 | ------- |
| 66 | Linoleic acid trimethylsilyl esterI | C_21_H_40_O_2_Si | FA | 28.22 | 3.84 | 0.77 |
| 67 | .(E)-9-Octadecenoic acid, TMS ester | C_21_H_42_O_2_Si | FA | 28.34 | 1.86 | 1.28 |
| 68 | 11-cis-octadecenoic acid 1TMS | C_21_H_42_O_2_S | FA | 28.44 | 0.66 | ------- |
| 69 | Stearic acid-monoTMS | C_21_H_44_O_2_Si | FA | 28.78 | 1.79 | 0.65 |
| 70 | Sucrose, octakis(trimethylsilyl) ether | C_36_H_86_O_11_Si_8_ | SU | 33.63 | 2.60 | ------- |
| 71 | D-(+)-Turanose, octakis(trimethylsilyl) ether | C_36_H_86_O_11_Si_8_ | SU | 34.44 | 3.79 | ------- |
| 72 | D-Turanose 7TMS | C_33_H_78_O_11_Si7 | SU | 35.31 | 2.92 | 0.68 |
| 73 | 1-Monolinoleoylglycerol trimethylsilyl ether | C_27_H_54_O_4_Si_2_ | FA | 35.62 | 1.54 | ------- |
| 74 | Linoleic acid, 2,3-bis-(O-TMS)-propyl ester | C_21_H_40_O_2_Si | FA | 38.53 | 1.41 | ------- |
| 75 | 1-Monolinoleoylglycerol trimethylsilyl ether | C_27_H_54_O_4_Si_2_ | FA | 39.82 | 0.75 | ------- |
|  |  |  |  |  | 97.54 | 99.82 |

RT: Retention Time; NC: Nitrogenous Compound; UH: Unsaturated hydrocarbon; AA: Amino Acid; OC: Organosilicon Compound; FA; Fatty Acid; OA: Organic Acid; SAL: Sugar Alcohol; **AL: Alcohol; ST: Steroid**; SU: Sugar; SA: Sugar Acid
